# Spatial and Temporal Variations of Six Criteria Air Pollutants in Fujian Province, China

**DOI:** 10.3390/ijerph15122846

**Published:** 2018-12-13

**Authors:** Weicong Fu, Ziru Chen, Zhipeng Zhu, Qunyue Liu, Cecil C. Konijnendijk van den Bosch, Jinda Qi, Mo Wang, Emily Dang, Jianwen Dong

**Affiliations:** 1 College of Landscape Architecture, Fujian Agriculture and Forestry University, Fuzhou 350002, Fujian, China; weicongfufj@163.com (W.F.); fjchenziru@126.com (Z.C.); zhuzhipeng512@126.com (Z.Z.); fafulqy@gmail.com (Q.L.); 2 Urban Forestry Research in Action, Department of Forest Resources Management, The University of British Columbia, Vancouver V6T 1Z4, BC Canada; cecil.konijnendijk@ubc.ca; 3 Collaborative for Advanced Landscape Planning, Faculty of Forestry, The University of British Columbia, Vancouver V6T 1Z4, BC, Canada; 4 Faculty of Forestry, The University of British Columbia, Vancouver V6T 1Z4, BC, Canada; emily.dang@hotmail.com; 5 Faculty of built environment, University of New South Wales, Sydney 2052, Australia; jinda.qi@student.unsw.edu.au; 6 College of Architecture & Urban Planning, Guangzhou University, Guangzhou 510006, Guangdong, China; landwangmo@outlook.com

**Keywords:** attainment rates, major air pollutants, meteorological factors, Western Taiwan Strait Economic Zone

## Abstract

Air pollution has become a critical issue in the urban areas of southeastern China in recent years. A complete understanding of the tempo-spatial characteristics of air pollution can help the public and governmental bodies manage their lives and work better. In this study, data for six criteria air pollutants (including particulate matter (PM_2.5_, PM_10_), carbon monoxide (CO), sulfur dioxide (SO_2_), nitrogen dioxide (NO_2_) and ozone (O_3_)) from 37 sites in nine major cities within Fujian Province, China were collected between January 2015 to December 2016, and analyzed. We analyzed the spatial and temporal variations of these six criteria pollutants, as well as the attainment rates, and identified what were the major pollutants. Our results show that: (1) the two-year mean values of PM_2.5_ and PM_10_ exceeded the Chinese National Ambient Air Quality Standard (CAAQS) standard I levels, whereas other air pollutants were below the CAAQS standard I; (2) the six criteria air pollutants show spatial variations (i.e. most air pollutants were higher in the city center areas, followed by suburban areas and exurban areas, except for O_3_; and the concentrations of PM_10_, PM_2.5_, NO_2_, O_3_ were higher in coastal cities than in inland cities); (3) seasonal variations and the no attainment rates of air pollutants were found to be higher in cold seasons and lower in warm seasons, except for O_3_; (4) the most frequently present air pollutant was PM_10_, with PM_2.5_ and O_3_ being the second and third most frequent, respectively; (5) all the air pollutants, except O_3_, showed positive correlations with each other. These results provide additional information for the effective control of air pollution in the province of Fujian.

## 1. Introduction

For the past few decades, air pollution has been widely investigated as it can impact people’s lives either directly or indirectly. As demonstrated by previous epidemiological studies, respiratory diseases are caused and exacerbated by poor air quality [1,2,3,4,5,6,7]. Studies have even found that poor air quality significantly affects people’s feelings and reduces their sensory experiences [8]. Moreover, air pollutants have a major impact on climate change [9,10,11] and visibility degradation [12,13,14,15], due to light scattering and light absorption by the air pollutants, e.g., PM_10_ and PM_2.5_. Therefore, air pollutants have attracted substantial attention across the world. Jeff et al. developed a model that describes regional and small-scale spatial and temporal gradients for the monthly mass concentrations of particulate matters (PMs) [16]. Kota et al. monitored and simulated the gaseous pollutants and PMs throughout 2015 in nine cities located in different areas of India [17]. Since long-term air pollutants data was limited, Wang et al. used the atmospheric visibility (AV) data, which is a proven indicator of air quality, to evaluate the air quality in different areas of the world. They found that in Europe and North America AV has increased, but in South and East Asia, South America, Australia, and Africa AV has decreased during the period from 1973 to 2007 [18]. Overall, previous studies have revealed that developed countries generally display higher air quality, whereas developing countries generally display poor air quality with substantially decreasing trends especially in China [16,17,18,19,20,21].

China, as the largest developing country, has experienced rapid economic growth in the past four decades. This has resulted in increased energy consumption, as well as increased air pollution [22,23,24,25,26,27,28]. To tackle this serious air quality issue, a series of laws and standards have been formulated by the Chinese government, e.g., Prevention and Control of Atmospheric Pollution (PCAP) and the Emission Standards of Air Pollutants for Thermal Power Plants (APTPP, GB13223-2003). In 2012, the State Council revised the Chinese National Ambient Air Quality Standards (CAAQS, GB3095-2012), which established the limit values of PM_10_, PM_2.5_ (particulate matter with aerodynamic diameter less than 2.5 μm), CO, SO_2_, NO_2_ and O_3_. Since then, CAAQS has become the authoritative standard for China’s air quality assessment. Analysis of the simultaneous characteristics of these six air pollutants can help the public, scientists and policy makers gain a comprehensive understanding of the air quality. Some studies have evaluated air quality according to CAAQS [29,30]. Ji et al. conducted an analysis of two regional events in northern China between October and November 2009, using PM_10_, SO_2_ and NO_x_ data collected in 24 monitoring sites [30]. Song et al. measured the Air Quality Index (AQI), as well as concentration of various air pollutants (PM_10_, PM_2.5_, SO_2_, NO_2_, CO, O_3_) during two episodes of Chinese New Year festivities in the city of Jinan [29]. However, due to the lack of high spatial-temporal resolution data (most monitoring site in China began collecting air pollutant data in January 2014, whereas some cities such as Putian and Nangping began collecting data in January 2015), not many studies have focused on all the six criteria air pollutants.

Fujian is a sub-tropical monsoon province in the coastal area of southeastern China. The province covers an area of 124,000 km^2^, and mountainous areas account for 89.3% of the province’s land area. In 2015, the population of Fujian was 38.39 million. Its industrial output was 438.88 billion Chinese Yuan (CNY, light industrial output 216.82 billion CNY, heavy industry output 222.06 billion CNY) and agricultural output was 371.78 million CNY. The number of vehicles in the province was 4.37 million. Fujian is considered the main economic driver for the Western Taiwan Strait Economic Zone (WTSEZ). Like the Beijing-Tianjin-Hebei (JTH), the Pearl River Delta (PRD) and the Yangtze River Delta (YRD), as it is supported by the Chinese central government. The recent rapid economic growth and urbanization has led to increasing conspicuous air pollution in Fujian Province [31]. Due to the hilly landforms of Fujian, coastal cities and inland cities are blocked by mountains, which results in differences of air quality between cities. Differences in socioeconomic factors (e.g., residents’ activities, industrial activities, urban size and urban greening) also cause variation in air quality in the city center, suburb and exurban areas. Understanding the temporal and spatial variation of the six pollutants is of great significance to the public and policy makers. Previous studies on air pollutants in Fujian province were mostly concentrated in Fuzhou city [32,33,34,35] and Xiamen city [36,37,38,39,40,41,42,43,44], and focused on the chemical composition and pollution sources of PMs. To date, studies on the temporal and spatial variation of air pollution with the province of Fujian as the study area have not been reported.

In this study, air pollutant data (e.g., PM_2.5_, PM_10_, CO, SO_2_, NO_2_ and O_3_) collected from 37 monitoring sites in nine major cities within the province of Fujian between 1 January 2015 to 31 December 2016 were investigated. The purposes of this study were to answer four questions: (1) to investigate whether there are temporal and spatial variations of air pollutants; (2) to study the measurement results of the monitoring sites in Fujian Province; (3) determine the hourly major pollutants that exceed the Chinese Ambient Air Quality Standards (CAAQS, GB3095-2012); (4) to examine the inter-correlation of the six criteria pollutants in the five regions based on two-year data and seasonal data, and the correlation between air pollutants and meteorological factors.

## 2. Materials and Methods

### 2.1. Data

**Figure 1:**
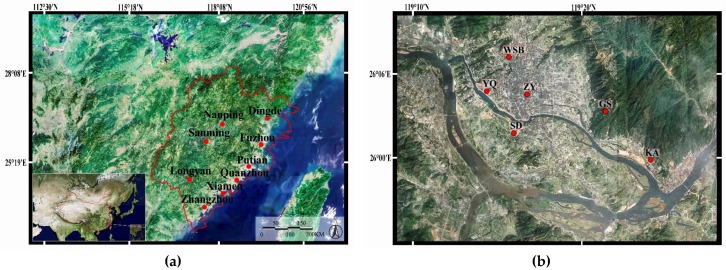

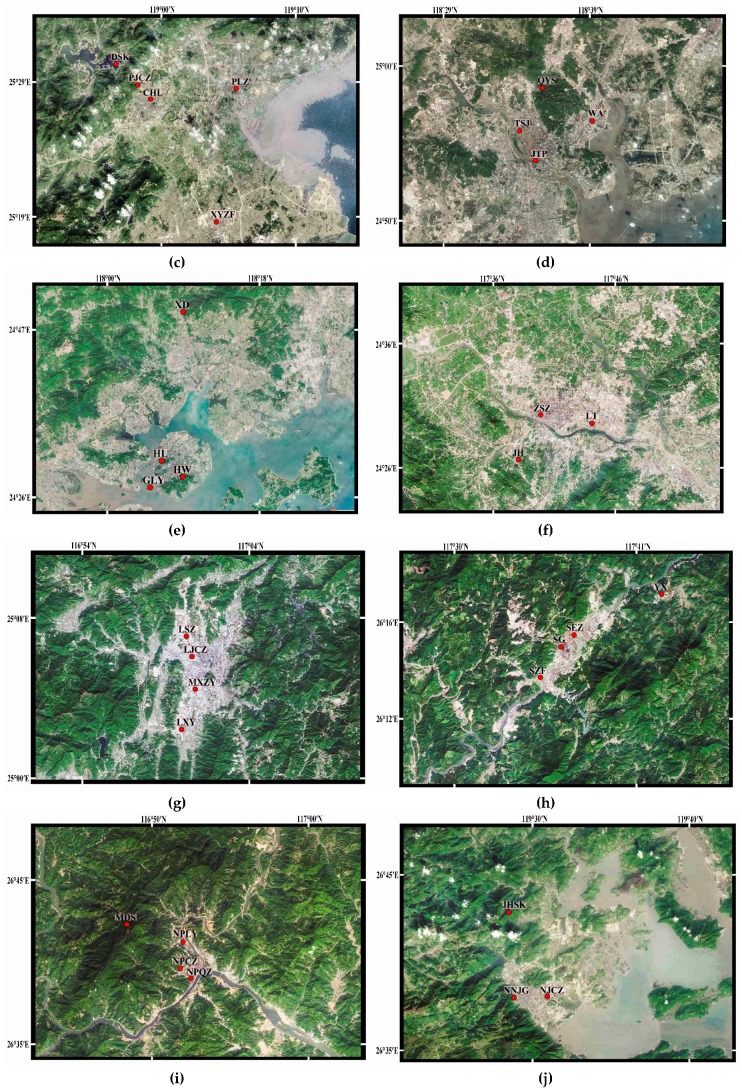
The locations of the 9 major cities in Fujian province (**a**) and the locations of the monitoring sites (**b**) in Fuzhou city; (**c**) in Putian city; (**d**) in Quanzhou city; (**e**) in Xiamen city: (**f**) in Zhangzhou city; (**g**) in Longyan city; (**h**) in Sanming city; (**i**) in Nanping city; and (**j**) in Ningde city.

Air pollutant data was obtained through the official release by the China National Environmental Monitoring Center (CNEMC, http://www.cnemc.cn/). The CNEMC is respected as an authority that develops the air pollutants information datasets in China. Multiple sites (3–6) were set up in each city as shown in Figure 1 and Table 1. The sites were designed as a mix of urban and background sites, with most of the sites in urban areas, and a few in suburban and exurban areas as background sites. These included 20 sites in the city central areas, eight in suburban areas and nine in exurban areas. At each monitoring site, automated monitoring systems were installed and measured the ambient air pollutant per hour automatically according to the standards set by China Environmental Protection Standards (CEPS, HJ 193-2013 and HJ 655-2013 http://www.es.org.cn/download/).

The automatic air pollutants monitoring system of PM_2.5_, PM_10_, NO_2_, SO_2_, O_3_ and CO consisted of the collection unit, the calibration device, the analytical unit, the measurement unit, the data collection and transport unit and other accessory equipment. The micro-oscillating balance method and the β-ray absorption method were used to measure the concentrations of PM_2.5_ and PM_10_. The chemiluminescence method was used to measure the concentrations of NO_2_, the ultraviolet fluorescence method was used to measure the concentrations of SO_2_, and the ultraviolet (UV)-spectrophotometry method was used to measure the concentrations of O_3_. The non-dispersive infrared (NDIR) and the gas filter correlation infrared absorption method were used to measure the concentrations of CO [45].

Meteorological hourly data was obtained through the official release by the U.S. National Oceanic and Atmospheric Administration (NOAA: https://www.climate.gov). This data included wind speed, wind direction, temperature, relative humidity, dew point temperature, air pressure and rainfall. The NOAA is respected as an authority that develops the data sets on meteorological information all over the world.

### 2.2. Method

Five statistical approaches were utilized in this study to investigate the spatial and temporal variations of the six pollutants in Fujian province between 1 January 2015 to 31 December 2016. The study’s statistical approaches are explained in Section 2.2.1, Section 2.2.2, Section 2.2.3, Section 2.2.4 and Section 2.2.5 respectively.

#### 2.2.1. Spatial Variations

To contrast the spatial difference of the air pollutants in the 37 monitoring sites and the nine cities of Fujian Province, CNEMC, CEPS and the locations of the monitor sites were used as a base to divide the 37 monitoring sites into three categories: urban areas (Central Business District, CBD), suburban areas (areas besides the CBD and exurban areas) and exurban areas, as shown in Table 1. We also divided the nine cities into two categories: inland cities and coastal cities. According to the geographical division based on whether the city is on the coastline, Longyan, Sanming and Nanping were defined as inland cities, while Ningde, Fuzhou, Putian, Quanzhou and Zhangzhou were defined as coastal cities. The locations of the nine major cities are shown in Figure 1. Analysis of Variance (ANOVA) was used to calculate whether or not there are significant differences of the air pollutants between the monitoring sites.

#### 2.2.2. Temporal Variations

To make the presentation of the temporal characteristics of the trends more concrete, the six air pollutants in the nine major cities were averaged monthly and seasonally (spring: March to May, summer: June to August, fall: September to November and winter: December to February) during the study period. We also used ANOVA to calculate the differences of the air pollutants between the months and seasons.

#### 2.2.3. Attainment Rate

The attainment rate is defined as the rates of hours where all the concentration of the six criteria air pollutants that are below the CAAQS Grade I standards. Attainment rates, which can easily reveal the information of air quality to the public, were calculated from all the monitoring sites in the province of Fujian. Then the data of different cities and different urban areas were divided to analyze the difference. ANOVA was used to define if there is a significant difference between the attainment rates of the monitoring sites.

#### 2.2.4. The Major Air Pollutants

Hourly ‘major air pollutants’ were identified to measure which pollutants contributed the most to air quality degradation. It is defined based on the Air Quality Index (AQI) system, using concentrations of individual pollutants. The pollutant which determined the maximum AQI is then defined as the major pollutant during that hour [46].

AQI is used to describe the air pollution level during a specific period such as 1 d or 1 h, ranging from 0 to 500. To calculate the AQI, the first step is to calculate the IAQIs for each of the six criteria air pollutants. The maximum value among IAQIs of the six air pollutants was defined as AQI. IAQI of the criteria air pollutants is calculated as follows:

(1) $${\mathrm{IAQI}}_{P}\;=\frac{{\mathrm{IAQI}}_{\mathrm{Hi}}{-\;\mathrm{IAQI}}_{\mathrm{Lo}}}{{\mathrm{BP}}_{\mathrm{Hi}}\;{-\;\mathrm{BP}}_{\mathrm{Lo}}}\;\left(C_{P}\;{-\;\mathrm{BP}}_{\mathrm{Lo}}\right){\;+\;\mathrm{IAQI}}_{\mathrm{Lo}}$$

where, IAQI_P_ and C_P_ are AQI and the concentration of one of the six criteria air pollutants (PM_2.5_, PM_10_, CO, SO_2_, NO_2_ or O_3_). BP_Hi_ and BP_Lo_ are the nearby value of the C_P_ (BP_Hi_ is the higher one, BP_LO_ is the lower one) (Table 1). IAQI_Hi_ and IAQI_Lo_ are corresponding IAQI of the BP_Hi_ and BP_Lo_ (Table 1).

#### 2.2.5. Correlations between Air Pollutants and Meteorological Factors

To gain a better understanding of the relationship between air pollutants and meteorological factors, we examined the relationship between the six air pollutants collected from 37 monitoring sites in nine cities within Fujian Province. Correlation analysis between the six air pollutants in coastal cities, inland cities, city central areas, suburban areas and exurban areas during the four seasons were conducted. The relationship between air pollutants and meteorological factors were also examined. For only three meteorological monitoring sites located in the city areas of Fujian Province (Fuzhou, Xiamen and Nanping), we performed correlation analyses in these three cities.

## 3. Results

### 3.1. Overview of the Air Pollutants

The two-year average concentrations of the six criteria pollutants collected from the 37 sites in the nine cities of Fujian Province are summarized in Table 2. The two-year mean concentrations of PM_2.5_ ranged from 11.93 (Mangdangshan) to 35.03 μg/m^3^ (Lantian). PM_2.5_ at all the sites, except Mangdangshan (MDS), exceeded the CAAQS Grade I standard of 15 μg/m^3^, but were below the Grade II standard of 35 μg/m^3^ in all sites, except Lantian (LT) where the concentration was 35.03 ± 21.36 μg /m^3^. The two-year average concentrations of PM_10_ ranged from 15.26 (MDS) to 64.04 μg/m^3^ (LT). PM_10_ in 17 of the 37 sites were below the Grade I standard (40 μg/m^3^); the other 20 of the 37 sites exceeded the Grade I standard (40 μg/m^3^) but met the Grade II standard (70 μg/m^3^). The tow-year concentrations of NO_2_ ranged from 3.45 (Mangdangshan) to 35.36 μg/m^3^ (Lantian), SO_2_ ranged from 4.07 (Mangdangshan) to 17.59 μg/m^3^ (Sangang), O_3_ ranged from 29.83 (Sanyangquzhengfu) to 75.85 μg/m^3^ (Gushan) and CO ranged from 0.41 (Xiuyuquzhengfu) to 1.45 mg/m^3^ (SanGang). The concentration of NO_2_, SO_2_, O_3_ and CO at all sites met the Grade I standard of NO_2_ (40 μg/m^3^), SO_2_ (20 μg/m^3^), O_3_ (80 μg/m^3^) and CO (4 mg/m^3^). Results showed that air quality is relatively good in the city areas of Fujian province.

### 3.2. Spatial Variations of Air Pollutants

Analysis of Variance (ANOVA) was used to calculate whether or not there are significant differences of the air pollutants between the monitoring sites. We analyzed the ANOVA of the tow-year mean concentrations of each air pollutants between all the 37 monitoring sites in Fujian Province. According the results of ANOVA, except for seven of the 582 pairs of monitoring sites, all the *p*-values were < 0.05, meaning that the air pollutant concentrations of the monitoring sites showed significant differences.

**Figure 2:**
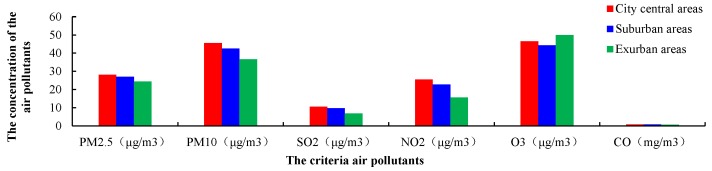
The average concentrations of the six criteria pollutants in three regional categories (city central areas, suburban areas and exurban areas) during 1 January 2015–31 December 2016.

As shown in Figure 2, according the CAAQS, the concentrations of PM_2.5_ in urban areas, suburban areas and exurban areas had all exceeded the Grade I standard, but were below the Grade II standard. The concentration of PM_10_ in urban areas and suburban areas exceeded the Grade I standard and were below the Grade II standard, the concentrations of PM_10_ in exurban areas were below the CAAQS Grade I standard. The concentrations of SO_2_, NO_2_, O_3_ and CO were below the CAAQS Grade I standard. The monitored air pollutants, except O_3_, showed significant spatial characteristics, such as the concentrations of air pollutants were highest in the city central areas, follow by the suburban areas and exurban areas. The concentration of CO in the suburban areas was higher than exurban areas, but only slightly higher than that in the city center. O_3_ is highest in the exurban areas, followed by the city center and suburban areas.

**Figure 3:**
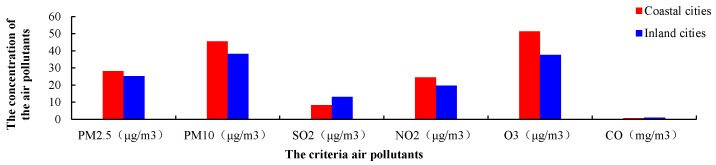
Two-year average concentrations of the six criteria pollutants in two topographic categories (coastal cities and inland cities).

Generally, the concentrations of PM_2.5_ and PM_10_ in the coastal cities and inland cities exceeded the Grade I standard and were below the CAAQS Grade II standard. The concentrations of SO_2_, NO_2_, O_3_ and CO in the coastal cities and inland cities were below the Grade I standard of CAAQS. All monitored air pollutants except SO_2_ and CO, were higher in the coastal cities than inland cities, (Figure 3). The average concentrations of SO_2_ were higher in inland cities (13.14 ± 13.27 μg/m^3^) than coastal cities (8.27 ± 8.57 μg/m^3^). Similarly, the average concentrations of CO were higher in inland cities (1.01 ± 0.66 mg/m^3^) than coastal cities (0.65 ± 0.32 mg/m^3^).

### 3.3. Temporal Variations of Air Pollutants

**Figure 4:**
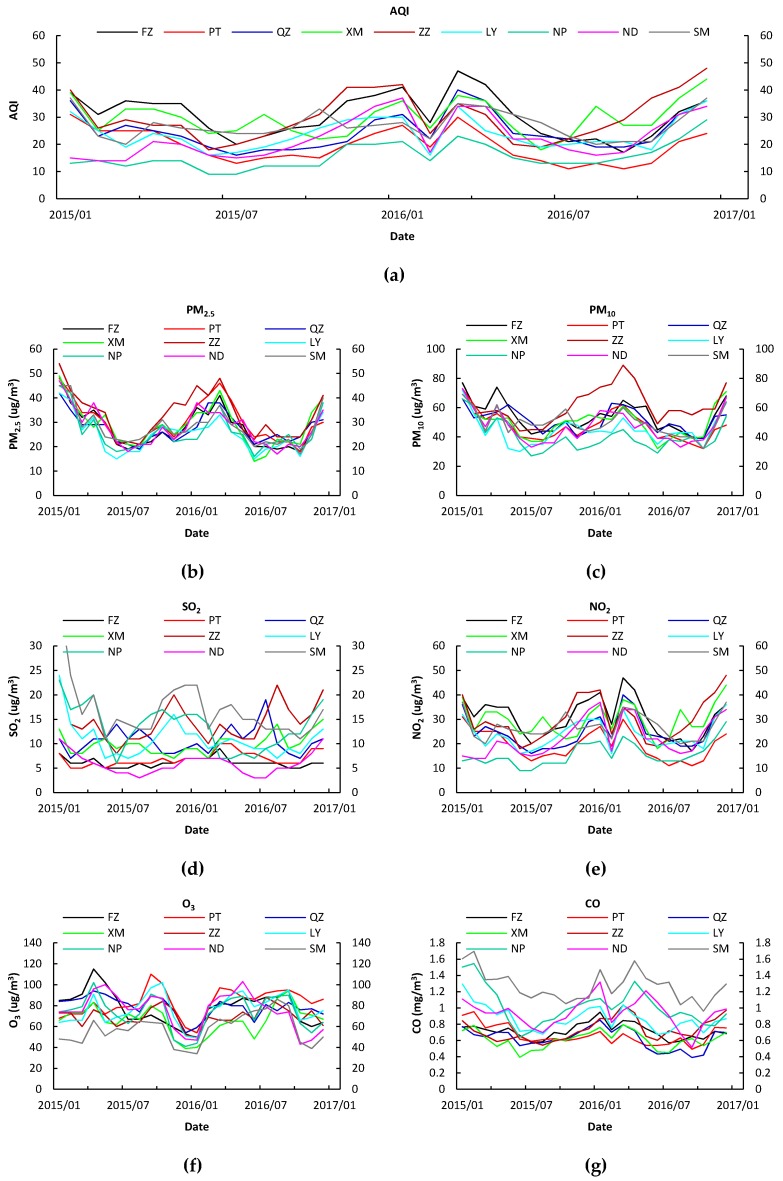
Temporal variation of the concentrations of six criteria air pollutants in the 37 stations in all 9 cities of Fujian province. (**a**) AQI, (**b**) PM_2.5_, (**c**) PM_10_, (**d**) SO_2_, (**e**) NO_2_, (**f**) O_3_ and (**g**) CO.

The six criteria air pollutants showed distinct seasonality and monthly, with all the ANOVA *p*-values < 0.01, meaning the air pollutants were significantly seasonal and monthly different. The seasonal concentration of PM_2.5_ and PM_10_ peaked in the winter (December–February), and decreased in spring (January–March), autumn (September–December) and summer (June–August) (Figure 2). Similarly, the seasonal variation of CO, NO_2_, and SO_2_ exhibited the highest in winter and spring and the lowest in summer and autumn (Figure 4). Distinct episodes where the concentration of SO_2_ and NO_2_ was relatively low occurred in February 2015 and February 2016. The explanation for this result to a large extent is that the longest holiday in China, the spring festival, occurred for about 10 days in February 2015 and February 2016. During the spring festival, most migrant workers return to their hometowns located in rural areas. In this time, the concentration of SO_2_ and NO_2_ decreased due to lower population density and traffic in the city, coupled with factory downtime. The concentration of O_3_ showed different seasonal patterns than the other air pollutants, where the highest concentration of O_3_ appeared in summer, followed by autumn, spring and winter.

### 3.4. Attainment Rate of Air Quality Standards

**Figure 5:**
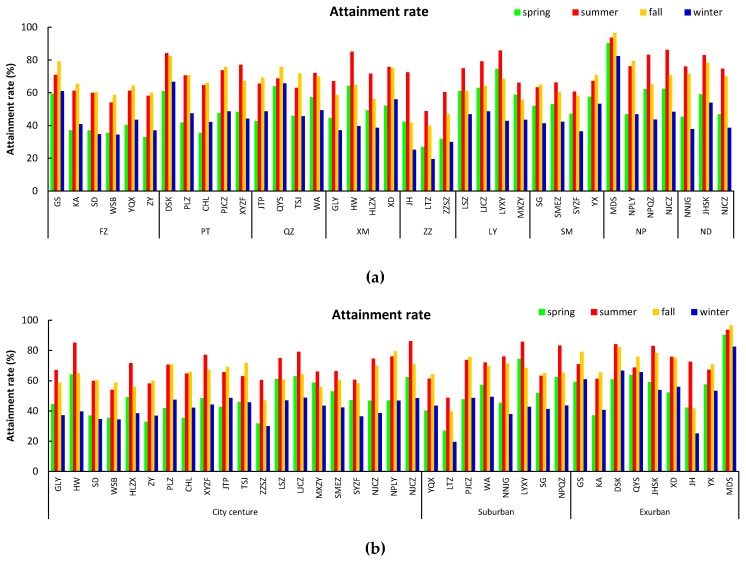
Seasonal variation of attainment rates (**a**) in the nine cities in Fujian province, (**b**) in the 37 stations classified into three region categories (city central areas, suburban areas and exurban areas).

Based on CAAQS, the attainment rates for all 37 monitoring sites showed temporal and spatial differences during the study period (Figure 5). Summer and autumn were the seasons with relatively high attainment rates, which were generally higher than 60%. Spring and winter were the seasons with relatively low attainment rate which were generally lower than 50%. The attainment rates of all nine cities were higher than 50%, except for Zhangzhou with a rate of 40.52% (Table 1). The attainment rates of the coastal cities were lower than the inland cities. The average attainment rate of the sites in the exurban area (65.87%) was higher than the city central area (56.82%) and suburban area (55.43%). The attainment rates in different areas of the cities were different—the attainment rate was highest in exurban areas follow by suburban areas and city central areas. The attainment rates also showed different seasonal variation in different areas of the urban areas—the highest season was summer in city central areas and suburban areas, but the highest seasons were summer and autumn in exurban areas. Sites in city central areas and suburban areas had strong seasonal variation, with the attainment rates in the summer (the highest) generally being more than one times higher compared to the attainment rates in winter (the lowest). However, sites in the exurban areas did not show such obvious seasonal variation.

### 3.5. The Major Air Pollutants

**Figure 6:**
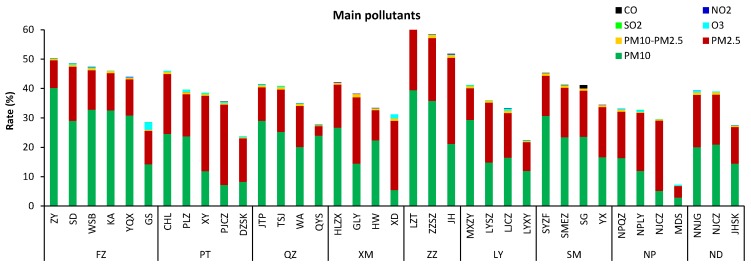
Total non-attainment rates and the major pollutants in the 37 stations of the 9 major cities in Fujian province (ranked by number of non-attainment days).

We illustrate the major air pollutants in all the non-attainment hours (defined as hours with any pollutant concentration exceeding CAAQS Grade I standards) in each site (Figure 4). Our results showed that PM_10_ was the most frequent major pollutant, PM_2.5_ being the second, and O_3_ being the third most frequent air pollutant collected at the 37 monitoring stations. Occurrences of CO, NO_2_, and SO_2_ as the major air pollutant were much less frequent (Figure 6). The rate of O_3_ as a major air pollutant was highest in GuShan and XiDong, which are located in exurban areas.

The frequency of the major air pollutants showed obvious seasonal variations. We report the fractions of each criteria air pollutant being the major pollutant in each season in Table 3 and Table 4. In summer and autumn, PM_10_ was the most frequent major pollutant in all five categories (city center, suburban, exurban, coastal city and inner city).

In winter, PM_2.5_ was the most frequent major pollutant, as hours where PM_2.5_ was the major pollutant account for over 30% in all categories except exurban areas with a rate of 25.11%. In spring, the situations were more complex. PM_2.5_ was the most frequent pollutant in city central areas and the inner cities. However, PM_10_ was the most frequent major air pollutant in suburban, exurban areas and coastal cities. O_3_ was the third frequent major pollutant, and was higher in the warm seasons (summer and autumn) and lower in cold seasons (spring and winter).

The frequency of the major air pollutants also showed spatial variations. The two-year frequency of the PMs was slightly different between city central areas (where the rates of PM_2.5_ and PM_10_ were 18.72% and 24.72% respectively) and suburban areas (where the rates of PM_2.5_ and PM_10_ were 19.19 and 22.66 μg/m^3^), which was higher than exurban areas (where the rates of PM_2.5_ and PM_10_ were 16.01% and 16.74%). The frequency of CO was higher in suburban areas than city central areas and exurban areas, and the frequency of CO was higher in inland cities than coastal cities. The frequency of O_3_ as the major air pollutants was higher in exurban areas than city central areas and suburban areas (Table 3). All six criteria air pollutants in coastal cities showed slightly higher frequencies than inland cities (Table 4).

### 3.6. Correlations between Air Pollutants and Meteorological Factors

Pearson correlation coefficients (R) were calculated between all the air pollutants in the six categories (i.e. all nine cities in Fujian Province, coastal cities, inland cities, city central areas, suburban areas and exurban areas of four seasons and yearly in Fujian Province, Table 5). Overall, significant positive correlation was found between the six criteria air pollutants (*p* < 0.01), except for the results with *p* value > 0.05 which was signed as NS. Over the study period, PM_2.5_ was relatively highly correlated with PM_10_, CO, SO_2_ and NO_2_ in all six categories. O_3_ was weakly correlated with other pollutants. The correlations among the six criteria air pollutants showed spatial variations. For instance, the correlations between PM_2.5_ and PM_10_ was highest in exurban areas, followed by suburban areas and city central areas (Table 5).

**Figure 7:**
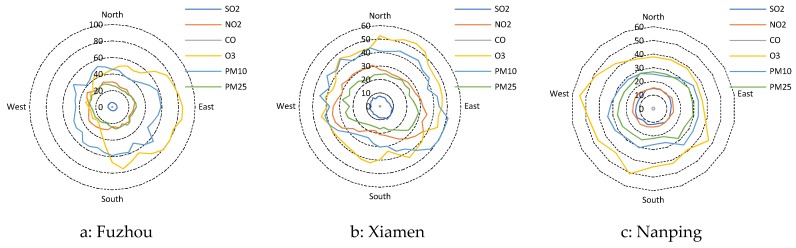
Mean mass concentrations of PM_2.5_, PM_10_, SO_2_, NO_2_, CO and O_3_ with respect to wind directions in (**a**) Fuzhou, (**b**) Xiamen and (**c**) Nanping, during 1 January 2015 and 31 December 2016.

Pearson correlation coefficients (R) between the air pollutants and the meteorological factors in Fuzhou, Xiamen and Nanping were also calculated (Table 6). Overall, the air pollutants, except O_3_, showed significant positive correlations with air pressure (AP), and significant negative correlations with wind speed (WS), temperature (T), dewpoint temperature (DPT), relative humidity (RH) and rainfall (RF) (*p* < 0.01, except for * means significant at 0.01 < *p* < 0.05, NS: no significance which had *p* > 0.05). O_3_ showed significant positive correlation with WS and T, and significant negative correlations with DPT, AP, RH and RF. As shown in Figure 7, wind directions were also considered in this study, and the results showed that to a large extent the wind from the areas with more population density and buildings have higher concentrations of air pollutants. The results of the correlation analysis indicated that rainfall scavenging was the most critical parameter in driving the periodic air pollutants cycle, which was different from that in the north China where the wind direction and speed played the major role.

## 4. Discussion

### 4.1. Fujian Province Has a Low Level of Air Pollution

The two-year mean concentrations of PM_2.5_ and PM_10_ collected from the 37 monitoring sites in Fujian province were 27.04 and 43.00 μg/m^3^, which both exceeded the CAAQS Grade I standard (15 μg/m^3^ for the annual mean value of PM_2.5_, and 40 μg/m^3^ for the two-year mean value of PM_10_). The two-year mean value of the concentrations of the other four criteria air quality were all below the CAAQS Grade I standard. Compared to the concentrations of the air pollutants in other regions of China, the concentration of air pollutants in the province of Fujian were relatively low [22,23,24,25,26,27,29,30,34,45]. It can be concluded that: (1) Fujian’s fossil fuel consumption was less than the other areas of China. In China, power generation mainly comes from fossil fuel consumption (69.6%), however, in Fujian the rate of fossil fuel consumption was 22.0% and the power generation mainly comes from hydropower, nuclear power (Chinese Yearbook, 2017) [31,39]; (2) all nine cities in Fujian are built along the river or along the coast (Figure 1), and Fujian province has to a subtropical climate with high wind speeds and high rainfall, therefore air pollutants are easily diluted [40,43]; (3) the forest coverage rate in Fujian Province is the highest (65.95%) in China (the forest coverage rate of China was 21.63% in 2016, Chinese Yearbook, 2017); (4) concentrations of the six criteria air pollutants show significant spatial characteristics.

### 4.2. Spatial Characteristics of the Six Criteria Air Pollutants

The concentrations of the six criteria air pollutants collected from the 37 monitoring sites showed significant spatial characteristics: the concentration of the six criteria air pollutants, except for O_3_, was highest in the city center areas than suburban and exurban areas, and the concentration of air pollutants, with the exception of CO and SO_2_, were higher in coastal cities than inland cities.

The concentrations of PM_2.5_, PM_10_ and NO_2_ were highest in the urban central areas followed by suburban areas and exurban areas. Residents’ activities (vehicle emission, cooking, fossil fuel consumption and so on) were the main factors affecting the spatial variation of the air pollutants [16,17,19]. In China, especially in the southeast areas, most residents live in the city center areas rather than the suburban and exurban areas. High population density causes a concentrated consumption of fossil fuel, cooking and vehicle emissions, which result in higher concentrations of PM_10_, PM_2.5_ and NO_2_ in the higher population density areas. From the data mentioned in Section 3.6 above, the R value was higher between PMs and NO2 than the R value between PMs and SO_2_ during the study period, emphasizing the importance of local vehicle exhaust emissions in the urban areas. Moreover, wind speeds in the city central areas are the lowest, followed by the suburban areas and exurban areas [23,25,26,28], which means the capacity of air pollutant diffusion is weakest in the city center areas, followed by the suburban areas and exurban areas.

The concentration of O_3_ was higher in exurban areas than the city center areas and suburban areas, with O_3_ concentration being slightly higher in the city center areas than suburban areas. O_3_ is a secondary pollutant formed by the photochemical reactions of NO_x_ and volatile organic compounds (VOCs) in the atmosphere and the formation rate of O_3_ also depends on the intensity of solar radiation [47]. O_3_ has a relatively long atmospheric residence time [46,47], and exurban areas are generally comprised of hills and mountainous areas which may affect the spread of O_3_, which may lead to higher concentrations of O_3_ in these areas.

The concentrations of CO and SO_2_, the main pollutants produced by industrial activities, were higher in inland cities than the coastal cities. Due to the geographical and political advantages, coastal cities are relatively more developed than inland cities. Highly developed areas such as coastal cities have more overall opportunities for people, which leads to higher population density and more residential activities (e.g., more vehicles, cooking, energy consumptions as well as a higher concentrations of pollutants). However, after 2010, strict environmental regulations, high labor costs and high pollution control fees in the coastal cities (Fujian Yearbook, 2016) such as Xiamen and Fuzhou, caused several companies to move their manufacturing bases from coastal cities to inland cities. As industrial activities are the main sources of CO and SO_2_ [31,42,44], such changes have led to higher concentrations of CO and SO_2_ in inland cities than in coastal cities.

### 4.3. Temporal Variation of the Six Air Pollutants

The concentrations of PM_10_, PM_2.5_, SO_2_, NO_2_ and CO showed similar seasonal variations, with the highest concentrations occurring in winter, followed by spring, autumn and summer (Figure 4). The seasonal variations reflect the effects of meteorological conditions (summer southwest monsoon and other season’s northeast monsoon) and man-made emissions. During the cold seasons (winter and spring), the air pollutants concentrations were relatively high may due to two factors: (1) stagnant meteorological conditions characterized by slow winds and shallow mixing layers occur more frequently, which trap the air pollutants near the surface, and lead to high air pollutant concentrations [35,36,48,49,50]. (2) Air pollutants from fossil fuel combustion sources, such as residential coal combustion for heating in northern China were brought into Fujian [35,36]. The sulfur content in the coal led to the release of SO_2_, which further enhanced the sulfate formation of secondary aerosols, which is the main contributor to PMs [48]. Biomass burning also contributes to the formation of high pollution in the winter, which has been revealed in previous studies [35,36,51,52]. The relatively higher air quality in warm seasons (summer and autumn) can be attributed to the following reasons: meteorological factors, such as stronger air convective mixing, higher WS, and faster vertical exchange; during this period air pollutants, especially PMs, SO_2_, NO_2_ and CO, may be sharply reduced by wet removal from the strong effects of large precipitation; and strengthened pollutant dilution from large scale transport due to East Asian summer monsoons. In the warm seasons, rainfall was also important for clearing the air, by driving the periodic air pollutants to scavenging cycle.

Distinct episodes where the concentration of SO_2_ and NO_2_ were relatively low were found in February 2015 and February 2016. The main explanation for this result is that the longest holiday in China, the spring festival, occurred for about 10 days in February 2015 and February 2016. During the spring festival, most migrant workers return to their hometowns located in rural areas. In this time, the concentration of SO_2_ and NO_2_ decreased due to lower population density and traffic in the city, coupled with factory downtime.

### 4.4. The Major Air Pollutant

We found that PMs were the most frequent major air pollutants in the province of Fujian. Previous studies done in Fujian Province found that vehicular exhaust was the primary contributor to atmospheric pollution in some cities with high PM_2.5_ concentrations. For example, Zhang et al. found that the impact of vehicle exhaust played a vital role in Fuzhou during 2007–2008 [52]. Wu et al. found that the significant enhancement of PM_2.5_ in urban areas in Fuzhou from 2007 to 2013 coincided with the growth of traffic pollutants followed by rapid urbanization [36]. O_3_ was the third most frequent major air pollutants in the Fujian Province. Previous studies revealed that industry and transportation sources played the most important role in the ozone formation and were the culprits of severe O_3_ pollution [46,47]. Policy makers and the public should pay more attention to vehicles as they are the primary cause of the major air pollutants in Fujian.

### 4.5. Correlation Analysis

Our results showed that the correlation between the six criteria air pollutants, except O_3_, showed significant positive correlations. Previous studies in China also showed similar results as our study [22,23,24,25,26,27]. However, in these previous studies the R value of the PMs between other air pollutants showed different results than ours. Our results showed that the R value between PMs and the other air pollutants were lower than previous studies taken in other areas of China [22,23,24,25,27]. This is because the formation of secondary PMs in southeast China was caused by higher organics mass concentrations, and were not only affected by exhaust of local pollutants, but also impacted by subtropical climate (high temperature, strong sunlight, and high photochemical activity) [51,52]. Weak negative correlations were observed between O_3_ and NO_2_, indicating the formation of secondary O_3_ and subtraction of NO_2_ by photochemical reactions simultaneously under favorable weather conditions.

Significant spatial variations were found, which means that different sources of air pollution produce different effects in different regions. The reasons for these results were similar to the result we have mention above (Section 4.1 and Section 4.2).

### 4.6. Limitations

This paper builds a platform for a comprehensive understanding of the spatial and temporal variation of the six criteria air pollutants collected from 37 monitoring sites in nine major cities within the province of Fujian. However, it should be noted that our study has limitations and further investigation is required. First, additional research is necessary to determine the effects of climate change on the air pollutants. Second, the analysis of air pollutants conducted in this study only included the data of the 37 sites in the nine major cities, as data was unavailable for the surrounding areas.

## 5. Conclusions

In this study, air quality data collected from 37 monitoring sites in nine major cities within the province of Fujian between 1 January 2015 to 31 December 2016 were analyzed. Since the monitoring stations are spread throughout the province, this study aimed to build a platform for a comprehensive understanding of the current air pollution in Fujian Province. We characterized the spatial and temporal variations of the concentrations of the six criteria pollutants, PM_2.5_, PM_10_, CO, SO_2_, NO_2_ and O_3_, as well as the air quality attainment rates and the major pollutants in each site. The results showed that: (1) air pollutants in the areas of Fujian Province were lower than most of the areas in China [22,23,24,25,26,27], however the concentrations of PM_2.5_ and PM_10_ exceeded the CAAQS Grade I standards; (2) the six criteria air pollutants in Fujian showed spatial variation (i.e., all six air pollutants, with the exception of O_3_, were highest in the city center areas, followed by suburban areas and exurban areas; and the concentrations of PM_10_, PM_2.5_, NO_2_, O_3_ were higher in the coastal cities than the inland cities); (2) temporal variations of air pollutants were also found (i.e., the concentrations of PM_2.5_, PM_10_, CO, SO_2_ and NO_2_, were highest in winter, followed by spring, autumn and summer; and the concentration of O_3_ was highest in summer, followed by autumn, spring and winter); (3) the attainment rates of the 37 monitoring sites also showed temporal and spatial variations; (4) we calculated the most frequent major air pollutants according to the CAAQA, and revealed that PM_10_ was the most frequent air pollutant, with PM_2.5_ and O_3_ being the second and the third, respectively.

This study also calls for future studies to investigate the associations between air quality and meteorological conditions, emissions in different areas, transportation and transformation of pollutants in both urban and rural areas. These analyses might further improve the understanding of the physical and chemical processes which affect air pollutants in the province of Fujian.

## Figures and Tables

**Table 1:** IAQI and the correspondingly limited concentrations of each pollutant (PM_2.5_, PM_10_, CO, SO_2_, NO_2_ and O_3_).

IAQI	PM_10_	PM_2.5_	SO_2_		NO_2_		CO		O_3_
	Daily	Daily	Daily	Hourly	Daily	Hourly	Daily	Hourly	Daily
0	0	0	0	0	0	0	0	0	0
50	50	35	50	150	40	100	2	5	160
100	150	75	150	500	80	200	4	10	200
150	250	115	475	650	180	700	15	35	300
200	350	150	800	800	280	1200	24	60	400
300	420	250	1600		565	2340	36	90	800
400	500	350	2100		750	3090	48	120	1000
500	600	500	2620		940	3840	60	150	1200

**Table 2:** Mean concentrations (mass ± standard deviation) of six criteria air pollutants and attainment rates (all the air pollutants below Grade I standards by CAAQS) in the 37 monitoring sites including 20 sites in city central areas (CCA), 8 in the suburban areas (SUA) and 9 in the exurban areas (EUA) in all the 9 major cities of Fujian Province, during 1 January 2015–31 December 2016.

City	Sites	Region Category	PM_2.5_	PM_10_	SO_2_	NO_2_	O_3_	CO	Attainment Rates (%)
			μg/m^3^	μg/m^3^	μg/m^3^	μg/m^3^	μg/m^3^	mg/m^3^	
Fuzhou (FZ)	Gushan (GS)	EUA	22.42 ± 17.59	31.22 ± 29.38	4.63 ± 3.91	14.69 ± 12.87	75.85 ± 42.80	0.56 ± 0.24	67.63
	Kuaian (KA)	EUA	27.16 ± 19.73	50.84 ± 36.58	6.46 ± 3.32	29.78 ± 20.97	46.22 ± 33.17	0.64 ± 0.26	51.18
	Shida (SD)	CCA	30.18 ± 20.69	48.45 ± 34.02	7.27 ± 4.41	30.68 ± 17.20	54.24 ± 35.61	0.69 ± 0.25	48.02
	Wusibeilu (WSB)	CCA	27.41 ± 20.29	51.26 ± 38.40	5.82 ± 3.84	33.08 ± 20.25	49.56 ± 36.05	0.69 ± 0.33	45.69
	Yangqiaoxilu (YQ)	SUA	26.19 ± 16.98	44.85 ± 34.52	5.36 ± 3.02	28.96 ± 17.08	50.56 ± 34.75	0.74 ± 0.24	52.41
	Ziyang (ZY)	CCA	26.42 ± 18.13	54.95 ± 36.23	5.44 ± 4.82	32.89 ± 19.72	47.58 ± 33.97	0.79 ± 0.29	47.04
	Average		26.77 ± 19.13	47.12 ± 35.82	5.85 ± 4.01	28.38 ± 19.26	53.90 ± 37.48	0.68 ± 0.28	52.00
Putian (PT)	Canghoulu (CHL)	CCA	30.94 ± 23.84	47.24 ± 33.70	5.23 ± 5.33	27.22 ± 14.93	47.05 ± 36.50	0.95 ± 0.43	52.09
	Jiancezhan (PJCZ)	SUA	30.43 ± 20.12	32.29 ± 27.69	3.60 ± 4.59	12.78 ± 10.10	55.91 ± 35.91	0.49 ± 0.30	61.50
	Liuzhong (PLZ)	CCA	26.90 ± 18.59	46.68 ± 29.18	8.23 ± 5.64	21.78 ± 15.39	59.83 ± 40.27	0.77 ± 0.32	57.68
	Xiuyuquzhengfu (XYZF)	CCA	31.58 ± 24.45	36.91 ± 31.80	10.63 ± 8.45	15.86 ± 11.20	64.58 ± 35.62	0.41 ± 0.24	59.26
	Dongzhenshuiku (DSK)	EUA	22.93 ± 19.07	28.51 ± 24.79	4.68 ± 6.26	11.67 ± 9.73	41.29 ± 30.67	0.64 ± 0.27	73.57
	Average		28.50 ± 21.65	38.23 ± 30.58	6.46 ± 6.71	17.82 ± 13.83	53.57 ± 36.98	0.65 ± 0.38	60.82
Quanzhou (QZ)	Tushanjie (TSJ)	CCA	29.47 ± 22.05	50.36 ± 46.37	12.28 ± 12.5	26.46 ± 14.87	54.91 ± 33.66	0.61 ± 0.26	56.65
	Jintoupu (JTP)	CCA	28.41 ± 21.30	52.14 ± 43.99	11.40 ± 12.0	26.55 ± 17.26	52.34 ± 36.13	0.66 ± 0.30	56.58
	Wanan (WA)	SUA	25.75 ± 17.23	43.69 ± 29.10	8.71 ± 8.79	24.46 ± 14.26	50.01 ± 32.25	0.63 ± 0.24	62.19
	Qingyuanshan (QYS)	EUA	19.95 ± 12.98	37.39 ± 32.62	8.28 ± 9.70	12.00 ± 8.86	52.78 ± 31.10	0.52 ± 0.26	68.57
	Average		25.83 ± 19.14	45.76 ± 39.17	10.13 ± 11.0	22.32 ± 15.41	52.34 ± 33.46	0.60 ± 0.27	61.00
Xiamen (XM)	Xidong (XD)	EUA	29.00 ± 20.64	41.93 ± 29.42	5.46 ± 5.00	13.15 ± 11.18	59.53 ± 39.78	0.48 ± 0.23	64.82
	Hongwen (HW)	CCA	24.36 ± 14.56	39.43 ± 30.79	7.63 ± 5.60	29.33 ± 18.17	45.30 ± 26.49	0.61 ± 0.22	63.51
	Gulangyu (GLY)	CCA	31.53 ± 22.18	49.14 ± 32.97	10.48 ± 12.7	27.60 ± 19.90	53.41 ± 28.07	0.62 ± 0.27	51.87
	Hulizhongxue (HL)	CCA	26.93 ± 18.77	44.64 ± 32.97	12.39 ± 8.97	33.42 ± 20.62	39.57 ± 27.82	0.57 ± 0.23	53.94
	Average		27.87 ± 19.34	43.65 ± 31.73	8.90 ± 8.92	25.81 ± 19.38	49.47 ± 31.94	0.57 ± 0.24	58.54
Zhangzhou (ZZ)	Lantian (LT)	SUA	35.03 ± 21.36	64.04 ± 36.10	16.1 ± 12.17	35.36 ± 17.02	42.61 ± 28.90	0.69 ± 0.30	33.79
	Zhangzhousanzhong (ZSZ)	CCA	32.07 ± 20.00	57.62 ± 36.36	13.91 ± 11.5	27.45 ± 17.19	43.03 ± 31.05	0.78 ± 0.38	42.30
	Jiuhu (JH)	EUA	33.87 ± 20.83	50.28 ± 32.96	11.59 ± 9.70	23.40 ± 19.02	50.86 ± 31.26	0.62 ± 0.23	45.47
	Average		33.63 ± 20.75	57.17 ± 35.60	13.82 ± 11.3	28.52 ± 18.46	45.57 ± 30.69	0.70 ± 0.32	40.52
Longyan (LY)	Longyanshizhuan (LSZ)	CCA	28.46 ± 19.09	41.41 ± 31.98	10.29 ± 8.05	17.31 ± 10.04	37.97 ± 29.28	0.83 ± 0.36	61.03
	Shijiancezhan (LJCZ)	CCA	25.70 ± 17.94	40.29 ± 28.21	11.83 ± 9.86	26.56 ± 15.88	48.05 ± 37.49	0.97 ± 0.44	63.77
	Minxizhiyejishuxueyuan (MXZY)	CCA	24.95 ± 17.46	48.37 ± 32.72	10.12 ± 8.96	27.05 ± 17.72	41.06 ± 36.93	0.85 ± 0.36	56.08
	Longyanxueyuan (LXY)	SUA	22.48 ± 16.08	36.63 ± 24.65	9.70 ± 4.85	18.83 ± 14.42	46.91 ± 34.71	0.71 ± 0.20	67.92
	Average		24.43 ± 18.23	40.11 ± 30.61	10.07 ± 8.40	21.63 ± 15.80	41.49 ± 35.35	0.81 ± 0.40	62.20
Sanming (SM)	Sangang (SG)	SUA	27.63 ± 17.85	44.64 ± 29.83	17.59 ± 17.1	27.91 ± 16.15	30.19 ± 28.17	1.45 ± 1.07	55.41
	Sanmingerzhong (SEZ)	CCA	28.24 ± 18.67	46.69 ± 32.54	17.32 ± 17.9	25.92 ± 14.23	31.20 ± 29.67	1.10 ± 0.62	55.55
	Sanyuanquzhengfu (SZF)	CCA	27.22 ± 18.79	50.95 ± 35.66	16.85 ± 14.9	27.75 ± 16.36	29.38 ± 30.71	1.28 ± 0.77	50.68
	Yangxi (YX)	EUA	27.23 ± 17.46	37.54 ± 29.29	12.00 ± 9.23	16.73 ± 9.77	35.30 ± 19.02	0.89 ± 0.50	62.24
	Average		27.58 ± 18.21	44.99 ± 32.31	15.96 ± 15.4	24.61 ± 15.10	31.50 ± 29.50	1.18 ± 0.80	55.97
Nanping (NP)	Nanpingshijiancezhan (NJCZ)	CCA	27.69 ± 19.80	29.07 ± 27.11	13.26 ± 11.1	15.98 ± 12.05	41.69 ± 32.82	1.02 ± 0.51	67.00
	Nanpinglvye (NPLY)	CCA	26.16 ± 21.06	33.34 ± 38.67	15.59 ± 14.1	15.06 ± 10.78	45.53 ± 35.79	0.94 ± 0.43	62.37
	Nanpingqizhong (NPQZ)	SUA	25.26 ± 17.90	37.48 ± 35.45	11.33 ± 8.83	16.19 ± 12.02	39.21 ± 35.45	0.98 ± 0.33	63.63
	Mangdangshan (MDS)	EUA	11.93 ± 11.95	15.26 ± 18.41	4.07 ± 5.37	3.45 ± 3.11	44.71 ± 46.27	0.59 ± 0.56	90.78
	Average		22.41 ± 15.45	28.60 ± 20.83	11.00 ± 6.80	12.56 ± 7.38	42.46 ± 25.81	0.88 ± 0.33	70.94
Ningde (ND)	Jinhanshuiku (JHSK)	EUA	24.09 ± 16.34	35.06 ± 27.73	4.33 ± 3.77	14.40 ± 11.42	44.22 ± 31.33	0.72 ± 0.33	68.58
	Jiaochengqunongjiju (NNJG)	SUA	28.21 ± 19.55	44.50 ± 30.98	7.11 ± 6.99	21.52 ± 15.86	47.73 ± 30.98	1.03 ± 0.47	57.72
	Ningdeshijiancezhan (NJCZ)	CCA	27.55 ± 19.52	44.35 ± 30.39	5.51 ± 4.50	24.74 ± 15.49	44.66 ± 36.32	0.85 ± 0.44	57.56
	Average		26.65 ± 18.65	41.38 ± 30.08	5.67 ± 5.42	20.28 ± 15.05	45.56 ± 35.36	0.87 ± 0.44	61.29
Fujian Province	Average		27.04 ± 19.85	43.00 ± 31.86	9.76 ± 8.66	22.44 ± 15.52	46.21 ± 32.95	0.77 ± 0.38	58.14

Note: The attainment rate is defined as hours with the concentration of all six criteria air pollutants below the CAAQS Grade I standards.

**Table 3:** The contributions of different air pollutants to the major pollutant (%) during the study period and the four seasons.

**City Center Areas**								
Pollutant	PM_2.5_	PM_10_	SO_2_	NO_2_	CO	O_3_	PM_10_-PM_2.5_	No attainment rates
Study period	18.72	24.72	0.01	0.02	0.00	0.31	0.77	44.55
Spring	24.05	27.13	0.01	0.04	0.00	0.34	0.96	52.53
Summer	7.16	22.81	0.01	0.01	0.00	0.48	0.40	30.87
Autumn	11.89	23.48	0.01	0.00	0.00	0.41	0.64	36.43
Winter	32.65	24.57	0.04	0.01	0.02	0.02	1.12	58.43
**Suburban Areas**								
Pollutant	PM_2.5_	PM_10_	SO_2_	NO_2_	CO	O_3_	PM_10_-PM_2.5_	No attainment rates
Study period	19.19	22.66	0.01	0.02	0.15	0.36	0.79	43.18
Spring	23.98	23.26	0.01	0.06	0.29	0.50	1.03	49.13
Summer	6.60	21.79	0.01	0.01	0.18	0.69	0.47	29.75
Autumn	12.68	21.48	0.00	0.00	0.02	0.27	0.51	34.96
Winter	34.58	23.32	0.02	0.00	0.06	0.01	1.17	59.16
**Exurban Areas**								
Pollutant	PM_2.5_	PM_10_	SO_2_	NO_2_	CO	O_3_	PM_10_-PM_2.5_	No attainment rates
Study period	16.01	16.74	0.01	0.01	0.00	0.79	0.55	34.11
Spring	21.23	18.50	0.02	0.00	0.00	1.41	0.71	41.87
Summer	7.85	15.51	0.01	0.00	0.00	1.01	0.36	24.74
Autumn	11.04	13.73	0.02	0.02	0.00	0.80	0.41	26.02
Winter	25.11	17.97	0.00	0.03	0.00	0.04	0.73	43.88

**Table 4:** The contributions of different air pollutants to the major pollutants (%) during the study period and the four seasons.

**Coastal Cities**								
Pollutant	PM_2.5_	PM_10_	SO_2_	NO_2_	CO	O_3_	PM_10_-PM_2.5_	No attainment rates
Study period	18.38	24.34	0.01	0.02	0.00	0.51	0.75	44.01
Spring	24.77	27.61	0.01	0.04	0.00	0.57	0.98	53.98
Summer	7.04	22.79	0.01	0.01	0.00	0.86	0.43	31.14
Autumn	11.37	21.83	0.01	0.01	0.00	0.58	0.54	34.34
Winter	31.19	24.20	0.01	0.02	0.01	0.03	1.07	56.53
**Inland Cities**								
Pollutant	PM_2.5_	PM_10_	SO_2_	NO_2_	CO	O_3_	PM_10_-PM_2.5_	No attainment rates
Study period	17.68	18.13	0.01	0.00	0.09	0.38	0.65	36.94
Spring	20.11	17.35	0.01	0.02	0.15	0.81	0.75	39.2
Summer	7.50	16.42	0.00	0.00	0.10	0.36	0.36	24.74
Autumn	13.24	17.75	0.01	0.00	0.01	0.37	0.57	31.95
Winter	31.20	19.68	0.47	0.01	0.07	0.00	0.93	52.36

**Table 5:** Correlations of pollutants in five regions based on the hourly data of the study period during 1 January 2015 to 31 December 2016.

	**All Cities in Fujian Province**					**Coastal Cities**					**Inland Cities**				
Pollutant	PM_10_	CO	SO_2_	NO_2_	O_3_	PM_10_	CO	SO_2_	NO_2_	O_3_	PM_10_	CO	SO_2_	NO_2_	O_3_
PM_2.5_	0.66	0.57	0.39	0.41	0.15	0.64	0.47	0.42	0.48	0.10	0.62	0.43	0.43	0.46	−0.02
PM_10_		0.47	0.38	0.49	0.14		0.38	0.42	0.54	0.07		0.40	0.40	0.45	−0.02
CO			0.23	0.37	−0.14			0.22	0.54	−0.14			0.44	0.44	−0.07
SO_2_				0.29	0.14				0.29	0.14				0.38	−0.6
NO_2_					−0.29					−0.38					−0.29
	**City Central Areas**					**Suburban Areas**					**Exurban Areas**				
Pollutant	PM_10_	CO	SO_2_	NO_2_	O_3_	PM_10_	CO	SO_2_	NO_2_	O_3_	PM_10_	CO	SO_2_	NO_2_	O_3_
PM_2.5_	0.64	0.39	0.37	0.43	0.03	0.65	0.28	0.30	0.40	0.13	0.70	0.38	0.34	0.48	0.17
PM_10_		0.32	0.37	0.47	0.05		0.28	0.34	0.49	0.12		0.31	0.39	0.51	0.21
CO			0.32	0.38	−0.23			0.41	0.34	−0.14			0.31	0.34	0.08
SO_2_				0.26	−0.11				0.32	−0.04				0.32	0.07
NO_2_					−0.32					−0.24					−0.13

**Table 6:** Correlations between air pollutants and meteorological factors based on the hourly data during 1 January 2015 to 31 December 2016.

Pollutant	Fuzhou						Xiamen						Nanping					
	WS	T	DPT	AP	RH	RF	WS	T	DPT	AP	RH	RF	WS	T	DPT	AP	RH	RF
PM_2.5_	**−0.18**	**−0.32**	**−0.35**	**0.29**	**−0.19**	**−0.20**	**−0.11**	**−0.34**	**−0.38**	**0.15**	**−0.15**	**−0.13**	**−0.16**	**−0.23**	**−0.29**	**−0.25**	**−0.13**	**−0.12**
PM_10_	**−0.16**	**−0.09**	NS	NS	**−0.18**	**−0.15**	**−0.06**	**−0.14**	**−0.29**	**0.13**	**−0.32**	**−0.21**	**−0.08**	**NS**	**−0.14**	**0.04**	**−0.28**	**−0.11**
CO	**−0.19**	**−0.20**	**−0.09**	**−0.10**	**−0.23**	−0.09 *	**−0.20**	**−0.21**	**−0.08**	**0.08**	**0.21**	NS	**−0.17**	**−0.25**	**−0.25**	**0.26**	NS	NS
SO_2_	NS	**−0.30**	**−0.39**	**0.36**	**−0.33**	**−0.09**	**−0.15**	**0.27**	**0.21**	**−0.08**	**−0.07**	**−0.16**	**−0.09**	**−0.34**	**−0.40**	**0.35**	**−0.13**	**−0.09**
NO_2_	**−0.34**	**−0.25**	**−0.15**	**0.15**	**0.19**	**−0.14**	**−0.41**	**−0.29**	**−0.13**	**0.07**	**0.25**	NS	**−0.21**	**−0.06**	**0.04**	**0.07**	**0.17**	NS
O_3_	**0.22**	**0.09**	**−0.10**	NS	**−0.49**	**0.09**	**0.27**	**0.32**	**0.07**	**−0.07**	**−0.40**	NS	**0.31**	**0.29**	**−0.10**	**−0.06**	**−0.68**	**0.11**

Note: The meteorological data include wind speed (WS), temperature (T), dewpoint temperature (DPT), relative humidity (RH), air pressure (AP) and rainfall (RF) were collected in the central urban areas in Fuzhou, Xiamen and Nanping. The Pearson correlation coefficients value in bold means the *p* value < 0.01 and with * means significant at *p* < 0.05, NS means no significant correlation.
